# Influence of Stress Jump Condition at the Interface Region of a Two-Layer Nanofluid Flow in a Microchannel with EDL Effects

**DOI:** 10.3390/nano13071198

**Published:** 2023-03-28

**Authors:** Muhammad Raees ul Haq, Ammarah Raees, Hang Xu, Shaozhang Xiao

**Affiliations:** 1 School of Computer Science and Software Engineering, Huaiyin Institute of Technology, Huaian 223003, China; 2 State Key Lab of Ocean Engineering, Collaborative Innovation Center for Advanced Ship and Deep-Sea Exploration (CISSE), School of Naval Architecture, Ocean and Civil Engineering, Shanghai Jiao Tong University, Shanghai 200240, China

**Keywords:** microchannel, nanofluid, stress jump condition, porous medium, electric double layer (EDL)

## Abstract

The influence of stress jump conditions on a steady, fully developed two-layer magnetohydrodynamic electro-osmotic nanofluid in the microchannel, is investigated numerically. A nanofluid is partially filled into the microchannel, while a porous medium, saturated with nanofluid, is immersed into the other half of the microchannel. The Brinkmann-extended Darcy equation is used to effectively explain the nanofluid flow in the porous region. In both regions, electric double layers are examined, whereas at the interface, Ochoa-Tapia and Whitaker’s stress jump condition is considered. The non-dimensional velocity, temperature, and volume fraction of the nanoparticle profiles are examined, by varying physical parameters. Additionally, the Darcy number, as well as the coefficient in the stress jump condition, are investigated for their profound effect on skin friction and Nusselt number. It is concluded that, taking into account the change in shear stress at the interface has a significant impact on fluid flow problems.

## 1. Introduction

Two-layer flow in a microchannel is essential in practical applications like crude-oil extraction, thermal insulation, solidification of castings, and several other geophysical applications. Another example is the design of micro-electromechanical systems (MEMS). In addition, fluid flow properties depict unusual behaviors in a microchannel compared to a macro-scale channel. Consequently, it is of significant importance to scientifically study the two-layer microchannel flow, particularly taking into account the possible effect of EDL. Due to this reason, many research studies have been conducted on flows through a microchannel, considering the electric double layer effects for Newtonian fluids [1,2,3,4], and non-Newtonian fluids [5,6,7,8,9,10]. However, most of the works mentioned above are connected to single-layer flow. The flow attributes of immiscible liquid are noticeable in the biochemical and biological investigation processes [11]. A laminar fluid interface is rendered when two or more immiscible liquids stream in microfluidic devices. In most cases, the influences of the fluid interface are noteworthy and cannot be neglected in the investigation of biological sample separation. Some research studies that have investigated this correlation include the work Gao et al. [12], who obtained theoretical and experimental results to investigate the two-fluid electro-osmotic flow in microchannels, but the Maxwell stress balance condition at the interface was not taken in account. Later, Gao et al. [13] modified the interface condition, by including the shear stress balances, that result in a jump at the interface resulting from the specific surface charge density. Some more exciting work [14,15,16] includes the investigation of two-layer microchannel flow along with the electro-osmotic effect, and using the shear stress balance interface condition. Recently, Niazi and Xu [17] used nanofluids to assess the electro-osmotic effect in two-layer microchannel flow. They used Buongiorno’s model [18] to construct a mathematical model, and obtained the analytic solutions for their problem. Mainly, they concluded that the flow behavior was altered dramatically in the presence of Brownian diffusion, thermophoresis diffusion, and viscosity. H. Tahir et al. [19] used the optimal homotopy approach, to analyze the performance of a hybridized two-phase ferromagnetic nanofluid of ferrite nanoparticles, and their effects on heat transmission in the flow of the hybrid nanofluid. Based on their investigation, it can be concluded that the thermophysical characteristics and Curie temperature with two or more ferrites suspended in two or more base fluids, can be enhanced. In-depth analysis by Hammad et al. [20], covers the numerous uses of nanofluids, as well as the implications of variables such as nanoparticle type and size, which may open up new prospects for commercial applications.

Porous media are also critical for exploring the applications described above. For example, thickening alloys do not have a eutectic composition, resulting in the separation of the frozen and liquid portions of the casting. In this instance, the partially frozen areas can be thought of as a porous medium with varying permeability. While porous media have been used for a wide range of commercial and geological purposes, there are opportunities to investigate alternative uses, particularly for energy systems, such as compact heat exchangers, heat pipes, electronic cooling, and solar collectors, by exploiting porous media. For certain applications, it is not necessary to entirely fill the system with the porous medium; partial filling is adequate. In comparison to a system that is totally filled with porous media, partial filling reduces the pressure drop. In addition, partial filling prevents contact between the porous material and the surface, reducing heat loss from the porous material to the surface. Such a criterion is necessary in a system where the primary objective is to improve the thermal coupling between the porous medium and fluid flow, and reduce the system’s high thermal coupling with the surrounding environment. For instance, the objective of Mohamad’s [21] solar air heater, was to increase the rate of heat transfer from the porous medium, which is heated by solar radiation, to air, while minimizing heat loss to the ambient environment. In addition, partial filling helps to decrease the pressure drop. A partial filling of a channel with porous media drives the flow to exit from the core area to the outer region, depending on the permeability of the medium. This decreases the thickness of the boundary layer and therefore increases the rate of heat transfer. The porous medium also alters the effective thermal conductivity and heat capacity of the flow, and the solid matrix increases the rate of radiative heat transfer in a gas-based system. Hence, increases in heat transfer occur through three mechanisms: flow redistribution, thermal conductivity adjustment, and medium radiative property modification. Beavers and Joseph [22] pioneered this type of study, by modeling flow in a porous material using Darcy’s law. The effects of the interfacial layer on fluid mechanics and heat transmission are discussed in further detail in [23,24]. These articles investigate non-Darcian effects in flow in porous media, via the Brinkman–Forchheimer-extended Darcy equation. In [24], the authors presented a precise solution for the flow field at the interface. The fluid layer is located among a semi-infinite porous object and an impermeable outer border, in their proposed model. Nield [25] demonstrated that velocity shear is continuous across the porous part of the contact. This is not always the case for solid sections, as the averaged velocity shears do not always coincide. Then, Kuznetsov [26], and Ochoa-Tapia and Whitaker [27,28], developed the strategy for comparing the Brinkman-extended Darcy law to Stokes’ equations, that need a discontinuity in the stress but retain continuity in the fluid flow. They determined that solving at the interface utilizing the Ochoa-Tapia and Whitaker conditions, resolves the over-determination problem demonstrated in Nield [25].

We intend to investigate fluid flow in a microchannel half filled with porous media, in light of the practical implications of two-layer fluid flow in a microchannel. In the region with a porous layer, the Brinkmann-extended Darcy’s law is used to mathematically predict fluid flow, whereas Buongiorno’s model is used in the other zone. For this topic, we used the stress jump boundary condition at the interface, which had been overlooked in prior research, as well as the impacts of the electric double layer (EDL) and magnetic field. Utilizing the interface stress jump condition, it is possible to correct for the overestimation of the physical parameters involved in the problem. The Darcy number and stress jump condition variations are critical in analyzing heat and mass transport in this two-layer fluid flow problem.

## 2. Problem Formulation

**Figure 1:**
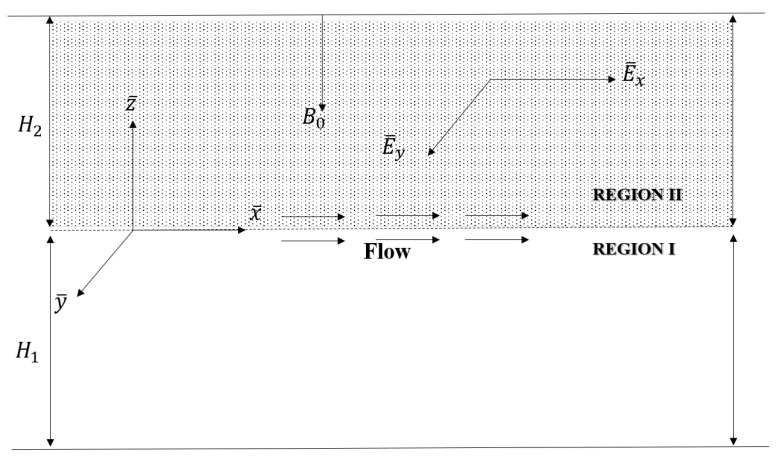
A schematic of the geometry.

We analyze the flow of an electro-osmotic fluid within a microchannel divided into two distinct regions (I and II). The elongated rectangular microchannel is horizontally positioned, with a width *W*, that is adequately greater than its height *H* (W/H>4; see Dauenhauer and Majdalani [29]). The length of the microchannel, *L*, is believed to be sufficient to prevent the apertures at the end from having an effect. $H_{1}+H_{2}=H$ are the height of the lower and upper layers, respectively. The interface among immiscible fluids is planar, based on the aforementioned assumptions. The parallel flow proposition can also be used to reduce the dimensions of the problem to two (2D). In Figure 1, the Cartesian coordinate system (x,y,z) is used, with *x* along the streamwise direction, *y* is parallel to the surfaces and normal to *x*, and *z* is perpendicular to the plates, parallel to each other. The lower and top walls have zeta potentials, temperature and nanoparticle volumes are represented as ${\bar{\zeta }}_{1},T_{w},C_{w}$ and ${\bar{\zeta }}_{2},T_{w},C_{w}$. Region I receives nanofluid containing Al_2_O_3_ nanoparticles, whereas Region II has porous media saturated with TiO_2_. Table 1 lists the physical parameters of the fluid and nanoparticles. The Buongiorno model is used to simulate nanofluid flow in Region I. The Brinkmann-extended Darcy law is employed to illustrate the flow of nanofluids in a porous layer region. The steady-state laminar flow is considered to be one-dimensional, owing to the significant presence of an electric field. due to the presence of an electric double layer (EDL) and the applied pressure.

The governing equations are modeled after the Navier-Stokes equations, with the driving force deriving from the electric and magnetic field, along with a pressure gradient. The mathematical models representing the physical phenomena in both the regions are as follows:

Region I:

(1) $$\begin{array}{cc} \nabla^{2}{\bar{\psi }}_{1}=- & \frac{{\bar{\rho }}_{e1}}{\varepsilon_{0}\varepsilon_{R_{1}}}, \end{array}$$

(2) $$\begin{array}{cc} \nabla \cdot V_{1}= & 0, \end{array}$$

(3) $$\begin{array}{cc} {\left(\rho_{1}\right)}_{f}\left(V_{1}\cdot \nabla \right)V_{1}= & -\nabla \bar{P}+\mu_{1}\nabla^{2}V_{1}+F_{1}, \end{array}$$

(4) $$\begin{array}{cc} {\left(\rho_{1}c_{p1}\right)}_{f}\left(V_{1}\cdot \nabla \right){\bar{T}}_{1}= & k_{nf1}\nabla^{2}{\bar{T}}_{1}+{\left(\rho_{1}c_{p1}\right)}_{s}[D_{B_{1}}\nabla {\bar{T}}_{1}\cdot \nabla {\bar{C}}_{1}\\ \; & +\frac{D_{T_{1}}}{T_{0}}\nabla {\bar{T}}_{1}\cdot \nabla {\bar{T}}_{1}]+\mu_{1}\bar{\Phi_{1}}, \end{array}$$

(5) $$\begin{array}{cc} \left(V_{1}\cdot \nabla \right){\bar{C}}_{1}= & D_{B_{1}}\nabla^{2}{\bar{C}}_{1}+\frac{D_{T_{1}}}{T_{0}}\nabla^{2}{\bar{T}}_{1}. \end{array}$$

Region II:

(6) $$\begin{array}{cc} \nabla^{2}{\bar{\psi }}_{2}=- & \frac{{\bar{\rho }}_{e2}}{\varepsilon_{0}\varepsilon_{R_{2}}}, \end{array}$$

(7) $$\begin{array}{cc} \nabla \cdot V_{2}= & 0, \end{array}$$

(8) $$\begin{array}{cc} \frac{{\left(\rho_{2}\right)}_{f}}{\epsilon^{2}}\left(V_{2}\cdot \nabla \right)V_{2}= & -\nabla \bar{P}+\frac{\mu_{2}}{\epsilon }\nabla^{2}V_{2}-\frac{\mu_{2}}{\kappa }V_{2}+F_{2}, \end{array}$$

(9) $$\begin{array}{cc} {\left(\rho_{2}c_{p2}\right)}_{f}V_{2}\cdot \nabla {\bar{T}}_{2}= & k_{nf2}\left(\nabla \cdot \nabla {\bar{T}}_{2}\right)+\epsilon {\left(\rho_{2}c_{p2}\right)}_{s}[D_{B_{2}}\nabla {\bar{T}}_{2}\cdot \nabla {\bar{C}}_{2}\\ \; & +\frac{D_{T_{2}}}{T_{0}}\nabla {\bar{T}}_{2}\cdot \nabla {\bar{T}}_{2}]+\bar{\Phi_{2}}, \end{array}$$

(10) $$\begin{array}{cc} \frac{1}{\epsilon }\left(V_{2}\cdot \nabla \right){\bar{C}}_{2}= & D_{B_{2}}\nabla^{2}{\bar{C}}_{2}+\frac{D_{T_{2}}}{T_{0}}\nabla^{2}{\bar{T}}_{2}. \end{array}$$

Here, ${\bar{\psi }}_{1}$ and ${\bar{\psi }}_{2}$ represent the dimensional electrostatic potentials in the two regions, and $\Phi_{1}$ and $\Phi_{2}$ are the viscous dissipation factor in two regions. The general forms of $\Phi_{1}$ and $\Phi_{2}$ are as follows:

(11) $$\begin{array}{cc} {\bar{\Phi }}_{1}= & 2\left({\left(\frac{\partial {\bar{u}}_{1}}{\partial \bar{x}}\right)}^{2}+{\left(\frac{\partial {\bar{v}}_{1}}{\partial \bar{y}}\right)}^{2}+{\left(\frac{\partial {\bar{w}}_{1}}{\partial \bar{z}}\right)}^{2}\right)+{\left(\frac{\partial {\bar{v}}_{1}}{\partial \bar{x}}+\frac{\partial {\bar{u}}_{1}}{\partial \bar{y}}\right)}^{2}+{\left(\frac{\partial {\bar{w}}_{1}}{\partial \bar{y}}+\frac{\partial {\bar{v}}_{1}}{\partial \bar{z}}\right)}^{2}\\ \; & +{\left(\frac{\partial {\bar{u}}_{1}}{\partial \bar{z}}+\frac{\partial {\bar{w}}_{1}}{\partial \bar{x}}\right)}^{2}-\frac{2}{3}{\left(\frac{\partial {\bar{u}}_{1}}{\partial \bar{x}}+\frac{\partial {\bar{v}}_{1}}{\partial \bar{y}}+\frac{\partial {\bar{w}}_{1}}{\partial \bar{z}}\right)}^{2}, \end{array}$$

(12) $${\bar{\Phi }}_{2}=\frac{\mu_{2}}{\kappa }V\cdot V+\mu_{eff}V\cdot \nabla^{2}V$$

### 2.1. Problem Statement and Assumptions

1. The direction of the flow is assumed to be along the *x*-axis.
2. The flow velocity in the $\bar{z}$-direction is negligible, since the length of microchannel *L* is much larger than its height *H*. Hence ${\bar{w}}_{i}\approx 0$,
3. The velocity component in the $\bar{y}$-direction is considered to be zero, i.e., ${\bar{v}}_{i}=0$,
4. The flow is assumed to uni-directional along the $\bar{x}$-axis but its properties changes with respect to the $\bar{z}$-axis, hence $V_{i}=\left({\bar{u}}_{i}\left(\bar{z}\right),0,0\right)$,
5. The body force, $F_{i}={æ}_{\mathrm{ei}}E+J_{i}\times B$, represents the sum of electro-osmosis and the electromagnetic forces, where $E=(E_{x},E_{y},0)$ is the electric field, $B=(0,0,B_{0})$ is the applied magnetic field, and $J_{i}=\sigma_{i}\left(E+V_{i}\times B\right)$ is the current density of the ion.
6. The inertial effects in the porous region of the microchannel (Region II) are negligible.
7. Region I of the channel is filled with nanofluid, while the channel’s Region II is filled with the porous medium saturated with nanofluid, having uniform permeability only.
8. Proceeding from the analysis presented in [26], the stress jump condition is utilized at the interface. Simultaneously, the electric potential, temperature, nanoparticle concentration, and flux at the interface are presumed to be continuous. Finally, the no-slip condition is applied to the velocity boundaries, while the temperature and nanoparticle concentration are assumed to have a constant distribution on the boundaries.

In light of the above assumptions, Equations (1)–(10) now take the form,

Region I: ($-H_{1}\leq \bar{z}\leq 0$)

(13) $$\begin{array}{cc} \frac{\partial^{2}{\bar{\psi }}_{1}}{\partial {\bar{z}}^{2}}= & -\frac{{\bar{\rho }}_{e1}\left(\bar{z}\right)}{\varepsilon_{0}\varepsilon_{R_{1}}} \end{array}$$

(14) $$\begin{array}{cc} \mu_{1}\frac{\partial^{2}{\bar{u}}_{1}}{\partial {\bar{z}}^{2}}= & \frac{\partial \bar{P}}{\partial \bar{x}}-E_{x}{\bar{\rho }}_{e1}-\sigma_{1}B_{0}\left(E_{y}-B_{0}{\bar{u}}_{1}\right) \end{array}$$

(15) $$\begin{array}{cc} {\bar{u}}_{1}\frac{\partial {\bar{T}}_{1}}{\partial \bar{x}}+{\bar{w}}_{1}\frac{\partial {\bar{T}}_{1}}{\partial \bar{z}}= & \alpha_{1}\frac{\partial^{2}{\bar{T}}_{1}}{\partial {\bar{z}}^{2}}+\tau_{1}[D_{B_{1}}\frac{\partial {\bar{T}}_{1}}{\partial \bar{z}}\frac{\partial {\bar{C}}_{1}}{\partial \bar{z}}\\ \; & +\frac{D_{T_{1}}}{T_{0}}{\left(\frac{\partial {\bar{T}}_{1}}{\partial \bar{z}}\right)}^{2}]+\frac{\mu_{1}}{{\left(\rho_{1}c_{p1}\right)}_{f}}{\left(\frac{\partial {\bar{u}}_{1}}{\partial \bar{z}}\right)}^{2} \end{array}$$

(16) $$\begin{array}{cc} {\bar{u}}_{1}\frac{\partial {\bar{C}}_{1}}{\partial \bar{x}}+{\bar{w}}_{1}\frac{\partial {\bar{C}}_{1}}{\partial \bar{z}}= & D_{B_{1}}\frac{\partial^{2}{\bar{C}}_{1}}{\partial {\bar{z}}^{2}}+\frac{D_{T_{1}}}{T_{0}}\frac{\partial^{2}{\bar{T}}_{1}}{\partial {\bar{z}}^{2}}. \end{array}$$

Region II: ($0\leq \bar{z}\leq H_{2}$)

(17) $$\begin{array}{cc} \frac{\partial^{2}{\bar{\psi }}_{2}}{\partial {\bar{z}}^{2}}= & -\frac{{\bar{\rho }}_{e2}\left(\bar{z}\right)}{\varepsilon_{0}\varepsilon_{R_{2}}}, \end{array}$$

(18) $$\begin{array}{cc} \mu_{eff}\frac{\partial^{2}{\bar{u}}_{2}}{\partial {\bar{z}}^{2}}= & \frac{\partial \bar{P}}{\partial \bar{x}}-E_{x}{\bar{\rho }}_{e2}-\sigma_{2}B_{0}\left(E_{y}-B_{0}{\bar{u}}_{2}\right)-\frac{\mu_{2}}{\kappa }{\bar{u}}_{2}, \end{array}$$

(19) $$\begin{array}{cc} \mu_{eff}\frac{\partial {\bar{T}}_{2}}{\partial \bar{x}}+{\bar{w}}_{2}\frac{\partial {\bar{T}}_{2}}{\partial \bar{z}}= & \alpha_{2}\frac{\partial^{2}{\bar{T}}_{2}}{\partial {\bar{z}}^{2}}+\tau_{2}[D_{B_{2}}\frac{\partial {\bar{T}}_{2}}{\partial \bar{z}}\frac{\partial {\bar{C}}_{2}}{\partial \bar{z}}\\ \; & +\frac{D_{T_{2}}}{T_{0}}{\left(\frac{\partial {\bar{T}}_{2}}{\partial \bar{z}}\right)}^{2}]+\frac{1}{{\left(\rho_{2}c_{p2}\right)}_{f}}\frac{\mu_{2}}{\kappa }{\bar{u}}_{2}^{2}, \end{array}$$

(20) $$\begin{array}{cc} {\bar{u}}_{2}\frac{\partial {\bar{C}}_{2}}{\partial \bar{x}}+{\bar{w}}_{2}\frac{\partial {\bar{C}}_{2}}{\partial \bar{z}}= & D_{B_{2}}\frac{\partial^{2}{\bar{C}}_{2}}{\partial {\bar{z}}^{2}}+\frac{D_{T_{2}}}{T_{0}}\frac{\partial^{2}{\bar{T}}_{2}}{\partial {\bar{z}}^{2}}. \end{array}$$

Here, $\alpha_{i}=k_{nf_{i}}/{\left(\rho_{i}c_{p_{i}}\right)}_{f}$ is the thermal diffusivity, with i=1,2 representing Region I and Region II, $\tau_{1}={\left(\rho_{1}c_{p_{1}}\right)}_{s}/{\left(\rho_{1}c_{p_{1}}\right)}_{f}$, $\tau_{2}=\epsilon {\left(\rho_{2}c_{p_{2}}\right)}_{s}/{\left(\rho_{2}c_{p_{2}}\right)}_{f}$ is the heat capacity ratio between the two regions of the microchannel, and $\mu_{eff}=\mu_{2}/\epsilon$, where ϵ is the porosity. The boundary conditions for the above stated governing equations in the two regions are as follows:

when $z=-H_{1}$:

(21) $${\bar{\psi }}_{1}={\bar{\zeta }}_{1},\;{\bar{u}}_{1}=0,\;{\bar{T}}_{1}=T_{w},\;{\bar{C}}_{1}=C_{w},$$

when z=0:

(22) $$\begin{array}{ccc} {\bar{\psi }}_{1}={\bar{\psi }}_{2},\;{\bar{u}}_{1} & = & {\bar{u}}_{2},\;{\bar{T}}_{1}={\bar{T}}_{2},\;{\bar{C}}_{1}={\bar{C}}_{2}, \end{array}$$

(23) $$\begin{array}{ccc} \varepsilon_{1}\frac{\partial \psi_{1}}{\partial \bar{z}}=\varepsilon_{2}\frac{\partial \psi_{2}}{\partial \bar{z}},\;\mu_{eff}\frac{\partial {\bar{u}}_{2}}{\partial \bar{z}}-\mu_{1}\frac{\partial {\bar{u}}_{1}}{\partial \bar{z}} & = & \frac{\beta \mu_{1}}{\sqrt{\kappa }}\bar{u_{2}},\;k_{nf_{1}}\frac{\partial {\bar{T}}_{1}}{\partial \bar{z}}=k_{nf_{2}}\frac{\partial {\bar{T}}_{2}}{\partial \bar{z}}, \end{array}$$

(24) $$\begin{array}{ccc} \frac{D_{T_{1}}}{T_{0}}\frac{\partial {\bar{T}}_{1}}{\partial \bar{z}}+D_{B_{1}}\frac{\partial {\bar{C}}_{1}}{\partial \bar{z}} & = & \frac{D_{T_{2}}}{T_{0}}\frac{\partial {\bar{T}}_{2}}{\partial \bar{z}}+D_{B_{2}}\frac{\partial {\bar{C}}_{2}}{\partial \bar{z}}, \end{array}$$

when $z=H_{2}$:

(25) $${\bar{\psi }}_{2}={\bar{\zeta }}_{2},\;{\bar{u}}_{2}=0,\;{\bar{T}}_{2}=T_{w},\;{\bar{C}}_{2}=C_{w}.$$

where β is the adjustable stress jump coefficient and κ is the permeability of the porous medium. The Poisson–Boltzmann equation [29], simplifies the relationship between the electrostatic potential ${\bar{\psi }}_{i}$, near the surface and the cumulative number of electrical charges for each unit of volume $\rho_{ei}$, at any point in the fluid.

(26) $$\frac{\partial^{2}{\bar{\psi }}_{i}}{\partial {\bar{z}}^{2}}=\frac{2n_{0}\hat{z}e}{\varepsilon_{0}\varepsilon_{R_{i}}}\sinh\left(\frac{\hat{z}e\bar{\psi_{i}}\left(\bar{z}\right)}{k_{B}\hat{T}}\right),$$

When the electrical potential is sufficiently small in comparison to the thermal energy of the ions, the Debye–Huckel linear approximation holds true, i.e., $|k_{B}\hat{T}\left|\ll \right|\hat{z}e{\bar{\psi }}_{i}\left(\bar{z}\right)|$, Equation (26) is reduced to

(27) $$\frac{\partial^{2}{\bar{\psi }}_{i}}{\partial {\bar{z}}^{2}}-\left(\frac{2n_{0}{\hat{z}}^{2}e^{2}}{\varepsilon_{0}\varepsilon_{R_{i}}k_{B}\hat{T}}\right){\bar{\psi }}_{i}=0,\;i=1,2.$$

### 2.2. Problem Non-Dimensionalization

To transform the governing equations to dimensionless forms, we introduce the following similarity transformations:

(28) $$\begin{array}{ccc} & & \eta =\frac{\bar{z}}{H},\;\Psi_{i}=\frac{\hat{z}e\bar{\psi_{i}}}{k_{B}\hat{T}},\;u_{i}=\frac{{\bar{u}}_{i}}{U_{ai}},\;\theta_{i}=\frac{{\bar{T}}_{i}-T_{0}}{T_{w}-T_{0}},\;\phi_{i}=\frac{{\bar{C}}_{i}-C_{0}}{C_{w}-C_{0}}, \end{array}$$

Substituting the non-dimensional variables defined in Equation (28), the fluid flow region is changed to $\left[-h_{1},h_{2}\right]$, with $h_{1}=H_{1}/H,h_{2}=H_{2}/H$, and the governing equations now take the form:

Region I $\left(-h_{1}\leq \eta \leq 0\right)$:

(29) $$\begin{array}{ccc} & & \Psi_{1}^{\prime \prime }-k_{1}^{2}\Psi_{1}=0, \end{array}$$

(30) $$\begin{array}{ccc} & & u_{1}^{\prime \prime }-Ha_{1}^{2}u_{1}+S_{e_{1}}Ha_{1}+\Gamma_{1}+k_{1}^{2}\Psi_{1}=0, \end{array}$$

(31) $$\begin{array}{ccc} & & \theta_{1}^{\prime \prime }+N_{B1}\theta_{1}^{\prime }\phi_{1}^{\prime }+N_{T1}{\left(\theta_{1}^{\prime }\right)}^{2}+Br_{1}{\left(u_{1}^{\prime }\right)}^{2}=0, \end{array}$$

(32) $$\begin{array}{ccc} & & \phi_{1}^{\prime \prime }+\frac{N_{T1}}{N_{B1}}\theta_{1}^{\prime \prime }=0. \end{array}$$

Region II $\left(0\leq \eta \leq h_{2}\right)$:

(33) $$\begin{array}{ccc} & & \Psi_{2}^{\prime \prime }-k_{2}^{2}\Psi_{2}=0, \end{array}$$

(34) $$\begin{array}{ccc} & & u_{2}^{\prime \prime }+\frac{1}{\gamma^{2}}\left(-Ha_{2}^{2}u_{2}+S_{e_{2}}Ha_{2}+\Gamma_{2}+k_{2}^{2}\Psi_{2}-\frac{1}{Da}u_{2}\right)=0, \end{array}$$

(35) $$\begin{array}{ccc} & & \theta_{2}^{\prime \prime }+N_{B2}\theta_{2}^{\prime }\phi_{2}^{\prime }+N_{T2}{\left(\theta_{2}^{\prime }\right)}^{2}+\frac{Br_{2}}{Da}u_{2}^{2}=0, \end{array}$$

(36) $$\begin{array}{ccc} & & \phi_{2}^{\prime \prime }+\frac{N_{T2}}{N_{B2}}\theta_{2}^{\prime \prime }=0. \end{array}$$

The corresponding boundary conditions are reduced to,

when $\eta =-h_{1}$:

(37) $$\Psi_{1}=\zeta_{1},\;u_{1}=0,\;\theta_{1}=1,\;\phi_{1}=1,$$

when η=0:

(38) $$\begin{array}{ccc} \Psi_{1}=\Psi_{2},\;\Psi_{1}^{\prime }=\lambda_{\varepsilon }\Psi_{2}^{\prime } & , & \;u_{1}=\frac{\lambda_{\varepsilon }}{\lambda_{\mu }}u_{2},\\ \gamma^{2}\lambda_{\varepsilon }u_{2}^{\prime }-u_{1}^{\prime }=\frac{\beta }{\sqrt{Da}}\frac{\lambda_{\varepsilon }}{\lambda_{\mu }}u_{2},\;\theta_{1}=\theta_{2} & , & \;\theta_{1}^{\prime }=\lambda_{nf}\theta_{2}^{\prime },\\ N_{B1}\left(\phi_{1}^{\prime }-\lambda_{D_{B}}\phi_{2}^{\prime }\right)+N_{T1}\left(\theta_{1}^{\prime }-\lambda_{D_{T}}\theta_{2}^{\prime }\right)=0 & , & \;\phi_{1}=\phi_{2} \end{array}$$

when $\eta =h_{2}$:

(39) $$\Psi_{2}=\zeta_{2},\;u_{2}=0,\;\theta_{2}=1,\;\phi_{2}=1.$$

where $\zeta_{i}=\hat{z}e_{0}{\bar{\zeta }}_{i}/\left(k_{B}\hat{T}\right)$ is the zeta potential, $k_{i}$ the electro-osmotic parameter.

(40) $$\begin{array}{ccc} & & k_{i}=\hat{z}eH\sqrt{\frac{2n_{0}}{\varepsilon_{0}\varepsilon_{Ri}k_{B}\hat{T}}},\;Ha_{i}=B_{0}H\sqrt{\frac{\sigma_{i}}{\mu_{i}}},\;S_{e_{i}}=\frac{E_{y}H}{U_{ai}}\sqrt{\frac{\sigma_{i}}{\mu_{i}}},\\ & & \Gamma_{i}=-\frac{H^{2}}{\mu_{i}U_{ai}}\frac{d\bar{P}}{d\bar{x}},\;U_{ai}=\frac{\varepsilon_{0}\varepsilon_{Ri}E_{x}k_{B}\hat{T}}{\mu_{i}\hat{z}e},\;Da=\frac{\kappa }{H^{2}},\;\gamma =\sqrt{\frac{\mu_{eff}}{\mu_{2}}}\\ & & N_{Bi}=\frac{\tau_{i}D_{Bi}\left(C_{w}-C_{0}\right)}{\alpha_{i}},\;N_{Ti}=\frac{\tau_{i}D_{Ti}\left(T_{w}-T_{0}\right)}{\alpha_{i}T_{0}},\\ & & Br_{i}=\frac{\mu_{i}U_{ai}^{2}}{k_{nfi}\left(T_{w}-T_{0}\right)} \end{array}$$

To measure the difference of physical properties, the following ratios are defined:

(41) $$\begin{array}{ccc} & & \lambda_{\varepsilon }=\frac{\varepsilon_{2}}{\varepsilon_{1}},\;\lambda_{nf}=\frac{k_{nf2}}{k_{nf1}},\;\lambda_{D_{B}}=\frac{D_{B2}}{D_{B1}},\;\lambda_{\mu }=\frac{\mu_{2}}{\mu_{1}}\\ & & \lambda_{\sigma }=\frac{\sigma_{2}}{\sigma_{1}},\;\lambda_{D_{t}}=\frac{D_{T2}}{D_{T1}},\;\lambda_{\alpha }=\frac{\alpha_{2}}{\alpha_{1}},\;\lambda_{\tau }=\frac{\tau_{2}}{\tau_{1}} \end{array}$$

where the physical parameters of the two regions are related as follows:

(42) $$\begin{array}{ccc} & & U_{a2}=\frac{\lambda_{\varepsilon }}{\lambda_{\mu }}U_{a1},\;S_{e_{2}}=\frac{\sqrt{\lambda_{\mu }\lambda_{\sigma }}}{\lambda_{\varepsilon }}S_{e_{1}},\;Br_{2}=\frac{\lambda_{\varepsilon }^{2}}{\lambda_{\mu }\lambda_{nf}}Br_{1},\;\Gamma_{2}=\frac{1}{\lambda_{\varepsilon }}\Gamma_{1},\\ & & k_{2}=\frac{1}{\sqrt{\lambda_{\varepsilon }}}k_{1},\;N_{B2}=\frac{\lambda_{\tau }\lambda_{D_{B}}}{\lambda_{\alpha }}N_{B1},\;N_{T2}=\frac{\lambda_{\tau }\lambda_{D_{T}}}{\lambda_{\alpha }}N_{T1},\\ & & Ha_{2}=\sqrt{\frac{\lambda_{\sigma }}{\lambda_{\mu }}}Ha_{1} \end{array}$$

The required ratios are calculated using the values from Table 1. Such as $\lambda_{nf}=1$, $\lambda_{\mu }=1$, $\lambda_{\alpha }=1$, and $\lambda_{\tau }=0.96$, and other ratios are chosen as $\lambda_{\sigma }=1.2$, $\lambda_{\varepsilon }=1.2$, $\lambda_{D_{B}}=1.2$, $\lambda_{D_{T}}=1.2$, and $\lambda_{\rho }=1$.

### 2.3. Skin Friction Coefficient and Nusselt Number

For the heat and mass transfer analyses we calculated the skin friction coefficient and Nusselt number as follows:

(43) $$C_{fi}=\frac{\tau_{wi}}{\frac{1}{2}\rho_{i}U_{ai}^{2}},\;Nu_{i}=\frac{H_{i}q_{wi}}{k_{nfi}\left(T_{w}-T_{0}\right)}$$

where i=1,2 denotes regions I and II, $\tau_{wi}$ denotes the shear stress, and $q_{wi}$ denotes the heat flux, which can be calculated using

(44) $$\tau_{wi}=\mu_{i}{\left.\frac{\partial \bar{u_{i}}}{\partial \bar{z}}\right|}_{\bar{z}={\left(-1\right)}^{i}H_{i}},\;q_{wi}=-k_{nfi}{\left.\frac{\partial \bar{T_{i}}}{\partial \bar{z}}\right|}_{\bar{z}={\left(-1\right)}^{i}H_{i}}.$$

Substituting Equations (28) and (44) into Equation (43), we get

(45) $$C_{fi}=\frac{2}{Re_{i}}u_{i}^{\prime }\left({\left(-1\right)}^{i}h_{i}\right),\;Nu_{i}=-\frac{h_{i}}{H}\theta_{i}^{\prime }\left({\left(-1\right)}^{i}h_{i}\right)$$

where $Re_{i}=\frac{H\rho_{i}U_{ai}}{\mu_{i}}$ is the Reynolds number. The relationship between two regions’ Reynolds numbers is defined by

(46) $$Re_{2}=\frac{\lambda_{\rho }\lambda_{\varepsilon }}{\lambda_{\mu }^{2}}Re_{1}$$

where $\lambda_{\rho }=\rho_{2}/\rho_{1}$.

## 3. Problem Solution

**Figure 2:**
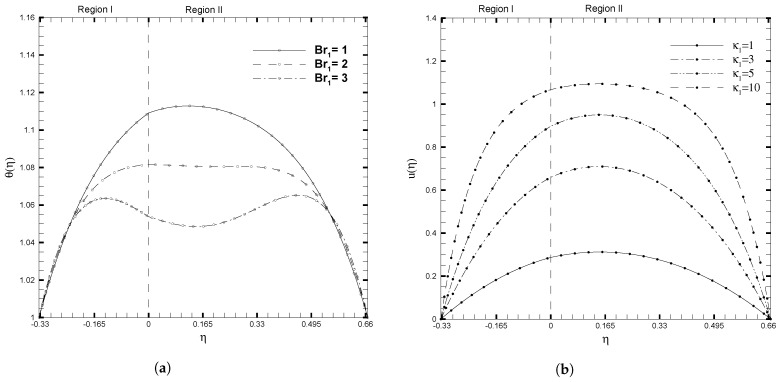
Comparison of solutions for (**a**) Temperature profile θ(η) with the variation in $Br_{1}$ and (**b**) Velocity profile u(η) with the variation in $\kappa_{1}$. Line: Niazi results [17]. Symbols: numerical results when β=0, Da=1, γ=1, $\zeta_{1}=\zeta_{2}=\kappa_{1}=1$, $H_{1}=1$, $H_{2}=2$, $Ha_{1}=S_{e_{1}}=\Gamma_{1}=1$, $Br_{1}=0.1$, and $N_{B1}=N_{T1}=0.1$.

Because Equations (29), (30), (33) and (34), are all second-order ODEs, they can be solved for their exact solutions. The Matlab built-in function dsolve is used to solve Equations (29), (30), (33), and (34), with respect to the boundary conditions Equations (37), (38) and (39) for $\Psi_{1}$, $u_{1}$, $\Psi_{2}$, and $u_{2}$. Because Equations (31), (32), (35), and (36) are nonlinear ODEs and exact solutions are difficult to obtain; thus, numerical simulations based on FDM are performed, as described in [30]. The nonlinearity of the equations is dealt with by Picard’s method. The iterative procedure stops once the following criterion is met.

(47) $$\frac{\sum_{j=1}^{N}\left|F^{i+1}\left(\eta \right)-F^{i}\left(\eta \right)\right|}{\sum_{j=1}^{N}\left|F^{i+1}\left(\eta \right)\right|}\leq 10^{-8},\;i\geq 1,$$

where *F* represents the field variables ψ, *u*, θ, and ϕ, and *N* represents the number of grid points. Here, $10^{-8}$ is the predefined tolerance error. To confirm our findings, we replicated those of Niazi and Xu [17], by setting β=0, Da=1, and γ=1. The comparisons for the velocity and temperature profiles are shown in Figure 2, which validates the results of the current problem. In this analysis, values of the parameters are selected based on the properties of the nanofluid given in Table 1. These values can vary depending upon the values of other parameters, to keep the system stable. For some values of parameters, such as β and $Se_{1}$, we have referred to the papers by Kuznetsov [26] and Niazi et al. [17].

**Figure 3:**
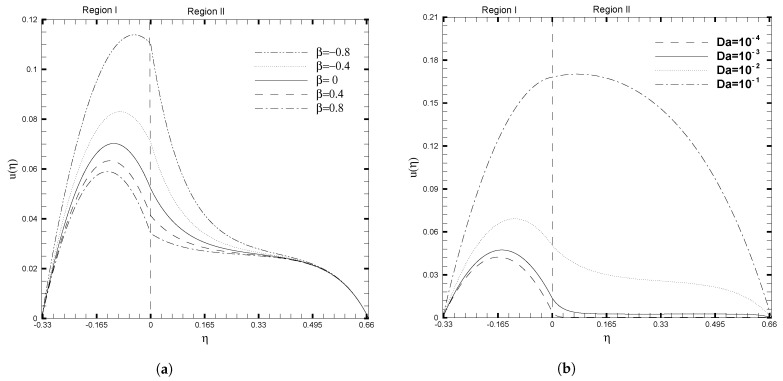
Velocity, u(η), for different values of stress jump coefficient (β) and Darcy number (Da), when $\zeta_{1}=\zeta_{2}=\kappa_{1}=1,H_{1}=1,H_{2}=2,Ha_{1}=S_{e_{1}}=\Gamma_{1}=1$, $Br_{1}=0.1$, and $N_{B1}=N_{T1}=0.1$.

Figure 3 illustrates the velocity profiles calculated for various different values of β and Da. The interface is located at η=0. According to the analysis in [26], we chose values for β that range between −0.8 and +0.8, and a Darcy number of the order of $10^{-1}$ or less. Figure 3a demonstrates that a change in stress can fundamentally alter the velocity profiles. When the stress at the interface increases, the slope of the tangent to the velocity distribution at η=0 changes dramatically. By gradually increasing the coefficient β, which accounts for the stress jump, the velocity is reduced noticeably. When β is negative, this apparent impact is particularly strong. Additionally, when the stress jump coefficient is varied, there is no change in the velocity profile near the upper wall. The Darcy number’s effect on the velocity profile is depicted in Figure 3b. The curve computed for $Da=10^{-2}$ contains three segments. One portion is contained within the momentum boundary layer adjacent to the boundary at η=−1, while the other portion is contained within the momentum boundary layer adjacent to the interface at η=0. As per the classical Darcy law, the fluid velocity increases inbetween two boundary layers, but stays unchanged in the porous layer. Additionally, as it enters the porous layer, the velocity decreases more rapidly in this third section. Similarly, the curves corresponding to $Da=10^{-3}$ and $Da=10^{-4}$ are almost identical; the difference is simply not visible due to the low velocity in the porous layer. There is no point on the curve $Da=10^{-1}$ where the velocity is constant. This is because, as the Darcy number increases, the width of the momentum boundary layers decreases.

**Figure 4:**
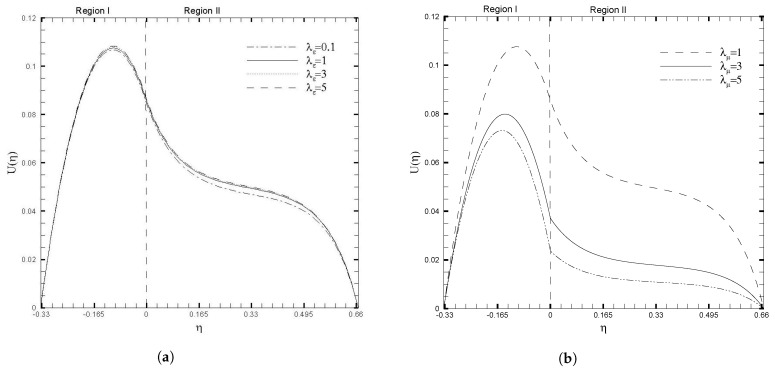
Velocity field, U(η), for different values of physical ratios $\lambda_{\varepsilon }$ and $\lambda_{\mu }$, when β=0.05, Da=0.01, γ=1, $\zeta_{1}=\zeta_{2}=\kappa_{1}=1$, $H_{1}=1$, $H_{2}=2$, $Ha_{1}=S_{e_{1}}=\Gamma_{1}=1$, $Br_{1}=0.1$, and $N_{B1}=N_{T1}=0.1$.

The influences of the physical ratios on the flow characteristics are displayed in Figure 4. It is observed in Figure 4a that, with the increase in the ratio of electric conductivity ($\lambda_{\varepsilon }$), the average velocity increases in Region I. At the same time, it decreases in Region II with a porous medium. The fluid in Region I conducts electricity better than in Region II. Further, in Figure 4b, an increase in the viscosity ratio ($\lambda_{\mu }$) decreases the velocity throughout the channel. In the case of a larger viscosity ratio, the velocity is smaller in Region I and Region II. The reason for these phenomena, is that when the viscosity ratio $\lambda_{\mu }>1$, the fluid viscosity in Region I is greater than that in Region II, resulting in a larger value of the average velocity in Region I.

**Figure 5:**
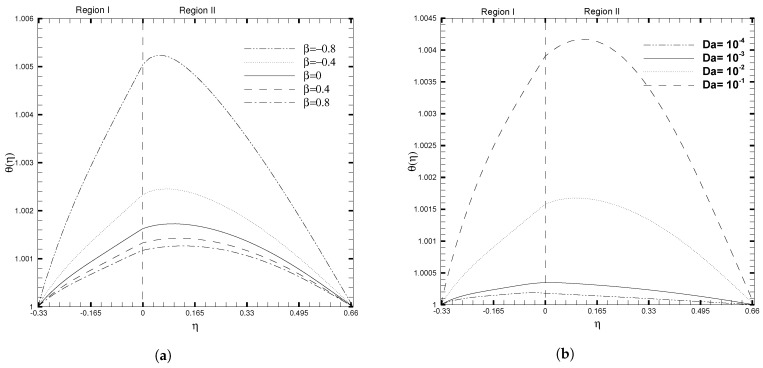
Temperature, θ(η), for different values of stress jump coefficient (β) and Darcy number (Da), when $\zeta_{1}=\zeta_{2}=\kappa_{1}=1$, $H_{1}=1,H_{2}=2$, $Ha_{1}=S_{e_{1}}=\Gamma_{1}=1$, $Br_{1}=0.1$, and $N_{B1}=N_{T1}=0.1$.

**Figure 6:**
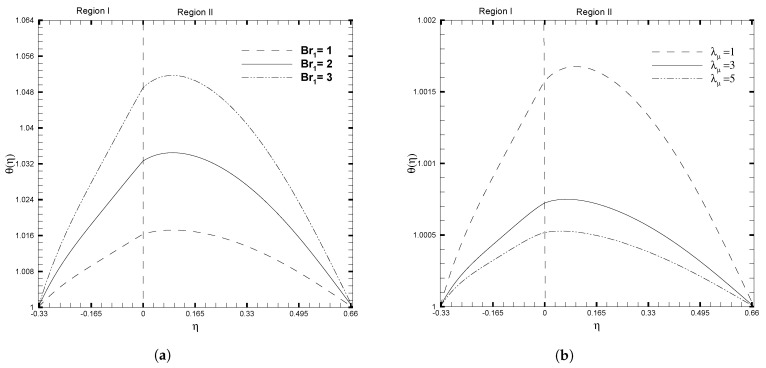
Temperature, θ(η), for different values of physical ratios $\lambda_{nf}$ and $\lambda_{\mu }$, when β=0.05, Da=0.01, γ=1, $\zeta_{1}=\zeta_{2}=\kappa_{1}=1$, $H_{1}=1,H_{2}=2$, $Ha_{1}=S_{e_{1}}=\Gamma_{1}=1$, $Br_{1}=0.1$, and $N_{B1}=N_{T1}=0.1$.

The significant influence of stress jump condition coefficient and Darcy number on the temperature profile was examined, and is shown in Figure 5. It can be observed that the temperature decreases throughout the channel for larger values of β, as displayed in Figure 5a. This figure demonstrates the significant effect of the stress jump on the non-dimensional temperature profile. A peak in the θ(η) is observed for a considerably smaller value of β=−0.8, and this curve flattens for a very large value of β. This illustrates that the increase in the stress jump coefficient reduces the temperature throughout the channel. While an adverse behavior in θ(η) is seen in the case of the Darcy number, as shown in Figure 5b. For lower values of the Darcy number, the temperature profile decreases significantly in the two regions. It is also observed that the temperature profile is indeed more significant in Region II than in Region I, which in turn depicts that the heat transfer rate is higher in the porous layer. Figure 6 shows the variation in the temperature profile for distinct values of the Brinkman number and viscosity ratio. An increase in Brinkman number $Br_{1}$, tends to increase the temperature profile, as in Figure 6a. A higher value of $Br_{1}$, slows the conduction of heat produced and hence the temperature rise is more considerable. The viscosity ratio shows an opposite trend on θ(η) as compared to the Brinkman number, as given in Figure 6b. As the value of the viscosity ratio, $\lambda_{\mu }$, increases, the temperature profile decreases throughout the channel. Physically, as the value of λμ increases, so does the amount of molecular conduction in the second region. As a result, the temperature of Region II decreases, which results in a decrease in the temperature throughout the channel, as illustrated in Figure 6b. However, when the viscosity ratio, $\lambda_{\mu }$, is minor ($\lambda_{\mu }\leq 1$), the position of the maximal value for θ(η) shifts towards Region II. For larger values of $\lambda_{\mu }$, the position shifts towards Region I. This shift occurs because the fluid interface must satisfy the boundary condition for continuous thermal flux.

**Figure 7:**
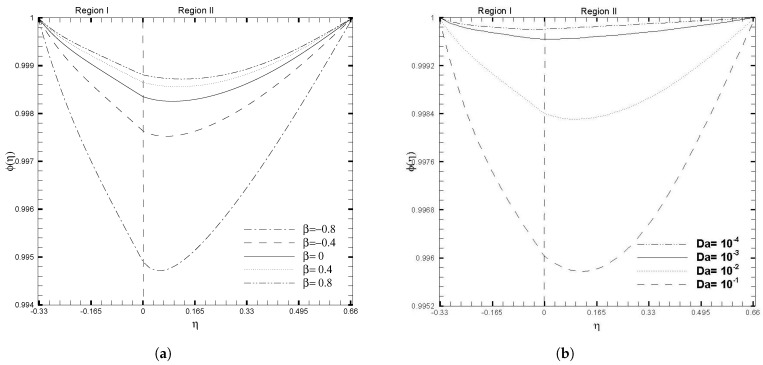
Concentration of nanoparticles, ϕ(η), for different values of adjustable coefficient in stress jump condition (β) and Darcy number (Da), when $\zeta_{1}=\zeta_{2}=\kappa_{1}=1$, $H_{1}=1$, $H_{2}=2$, $Ha_{1}=S_{e_{1}}=\Gamma_{1}=1$, $Br_{1}=0.1$, and $N_{B1}=N_{T1}=0.1$.

**Figure 8:**
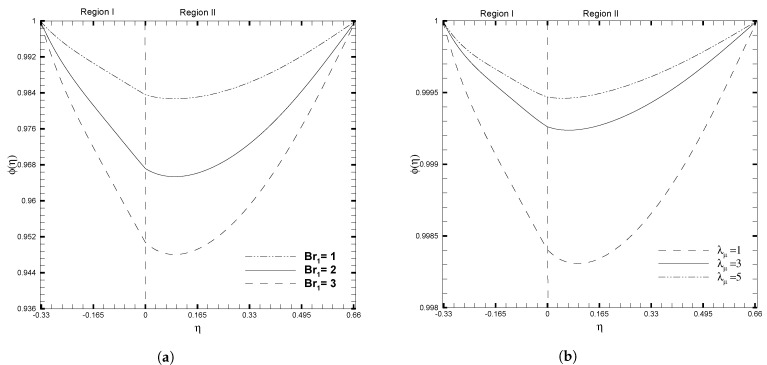
Concentration of nanoparticles, ϕ(η), for different values of physical ratios $\lambda_{nf}$ and $\lambda_{\mu }$, when β=0.05, Da=0.01, γ=1, $\zeta_{1}=\zeta_{2}=\kappa_{1}=1$, $H_{1}=1$, $H_{2}=2$, $Ha_{1}=S_{e_{1}}=\Gamma_{1}=1$, $Br_{1}=0.1$, and $N_{B1}=N_{T1}=0.1$.

Figure 7 illustrates the evolution of the nanoparticle volume fraction ϕ(η) as the stress jump coefficient (β) and Darcy number (Da) increase. As shown in Figure 7a, the volume fraction of nanoparticles decreases rapidly as β decreases, particularly for negative values of β. Nevertheless, an adverse behavior is seen in the ϕ(η) profile for the case of the Darcy number, as given in Figure 7b. The increase in Darcy number causes a significant decrease in the nanoparticle profile. In contrast, smaller values of the Darcy number have a minimal impact on the ϕ(η) and give almost a flat curve for $Da=10^{-3}$ and $Da=10^{-4}$. The impacts of the physical ratios and Brinkman number on the nanoparticle volume fraction, are shown in Figure 8. It is observed that the influence of $Br_{1}$ and $\lambda_{\mu }$ on ϕ(η), show opposite trends. Figure 7a shows that the ϕ(η) decreases as the Brinkman number increases, due to the increased fluid viscosity; while increasing the value of $\lambda_{\mu }$ accelerates the movement of nanoparticles toward the upper wall, this results in a decrease in the nanoparticle volume fraction, as illustrated in Figure 8b.

**Figure 9:**
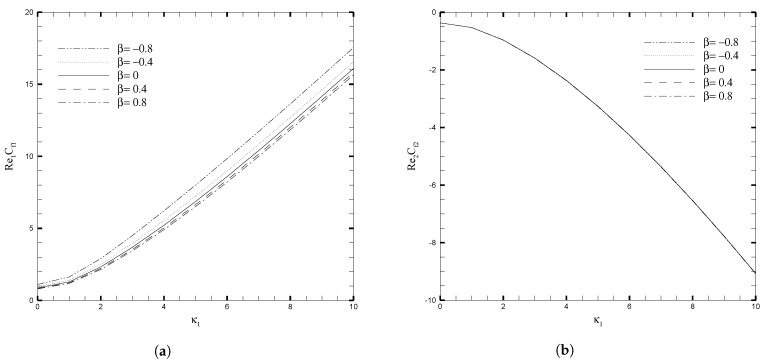
Local skin friction coefficient for different values of adjustable coefficient in stress jump condition (β), when Da=0.01, $\zeta_{1}=\zeta_{2}=\kappa_{1}=1$, $H_{1}=1$, $H_{2}=2$, $Ha_{1}=S_{e_{1}}=\Gamma_{1}=1$, $Br_{1}=0.1$, and $N_{B1}=N_{T1}=0.1$.

**Figure 10:**
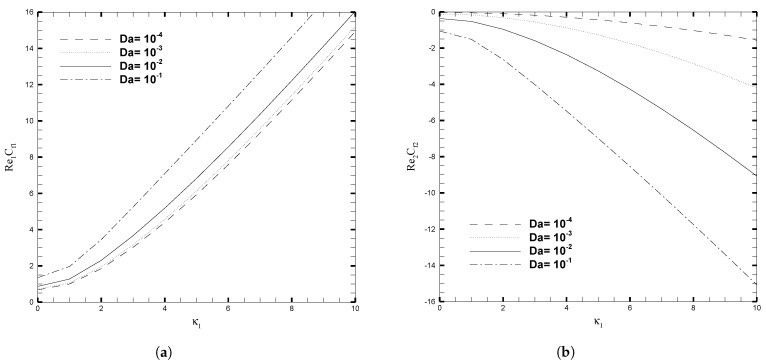
Local skin friction coefficient for different values of Darcy number (Da), when β=0.05, $\zeta_{1}=\zeta_{2}=\kappa_{1}=1$, $H_{1}=1,H_{2}=2$, $Ha_{1}=S_{e_{1}}=\Gamma_{1}=1$, $Br_{1}=0.1$, and $N_{B1}=N_{T1}=0.1$.

**Figure 11:**
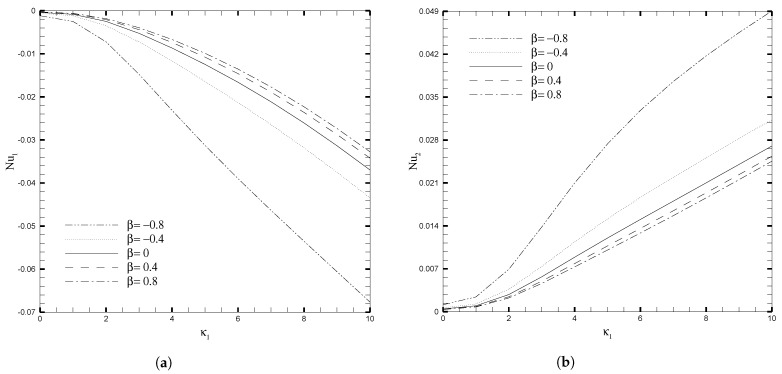
Nusselt number for different values of adjustable coefficient in stress jump condition (β), when Da=0.01, $\zeta_{1}=\zeta_{2}=\kappa_{1}=1,H_{1}=1,H_{2}=2,Ha_{1}=S_{e_{1}}=\Gamma_{1}=1$, $Br_{1}=0.1$, and $N_{B1}=N_{T1}=0.1$.

**Figure 12:**
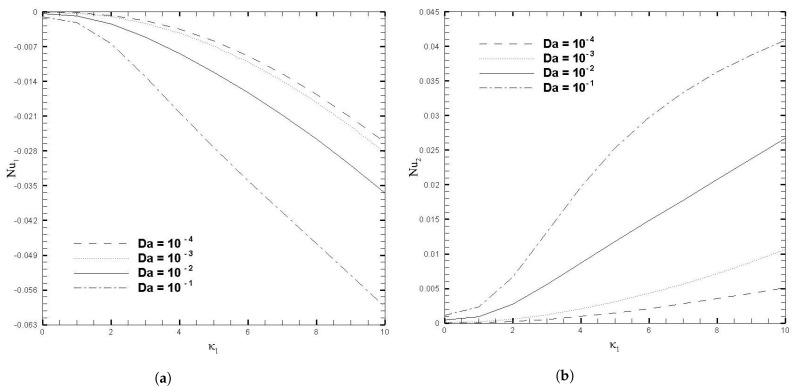
Nusselt number for different values of Darcy number (Da), when β=0.05, $\zeta_{1}=\zeta_{2}=\kappa_{1}=1$, $H_{1}=1$, $H_{2}=2$, $Ha_{1}=S_{e_{1}}=\Gamma_{1}=1$, $Br_{1}=0.1$, and $N_{B1}=N_{T1}=0.1$.

The variations in skin friction coefficient, $C_{fi}$, with $\kappa_{1}$, β, and Da are presented in Figure 9 and Figure 10, respectively. Since it has hitherto been observed that the β and Da produce a significant effect on the velocity profile, given in Figure 3, this pattern also holds true for the fluctuation of $C_{fi}$, but the orientation on the upper wall is the inverse of the direction on the lower wall. When β<0, the local skin friction ($C_{f1}$) rises, for increasing values of $\kappa_{1}$, and this increase becomes increasingly evident for larger values of $\kappa_{1}$. On the other hand, for β≥0, there is a slight increase in skin friction ($C_{f1}$) for larger values of $\kappa_{1}$ but for smaller values of $\kappa_{1}$ this increase is negligible. At the upper wall, variation in the β parameter has no effect on the skin friction coefficient ($C_{f2}$) when the electro-osmotic parameter is changed. Figure 10 illustrates the increase in the skin friction coefficient on the lower wall and the decrease on the upper wall, as $\kappa_{1}$ increases. This is because the coordinates are set in such a way that at the interface between the two layers, the signs on the top and bottom walls are reversed. An increase in the Darcy number increases the $C_{f1}$ significantly, but for smaller values of the Darcy number, this increase is not prominent. While at the upper wall, the decrease in $C_{f2}$ is evident for the increasing values of Darcy number. The influence of Nusselt number ($Nu_{i}$) with β and Da, for several values of $\kappa_{1}$, are shown in Figure 11 and Figure 12, respectively. As can be seen from these figures, increasing the value of $\kappa_{1}$ decreases the Nusselt number on the top wall, while increasing it on the bottom wall. Physically, the increase in $\kappa_{1}$ reduces the EDL effect, which leads to the enhancement of the fluid motion. Thus, it causes more heat conduction than heat convection, causing the decrease in the Nusselt number at the upper wall. On the other hand, for larger values of β and Da, the effect on $Nu_{i}$ is more evident, both at the upper and lower wall. As a result, calculations that do not take the increase in stress into account, may suffer a significant loss of accuracy.

## 4. Conclusions

In the EDL effect, physical analysis was performed on two-layer nanofluids in a microchannel partially filled with porous medium. The mathematical model for the two-layer nanofluid flow is developed using Buongiorno’s model. At the interface, Ochoa-Tapia and Whitaker’s proposed jump boundary condition is used, to map the Brinkman-extended Darcy equation to the Stokes’ equation. The finite difference method is employed to solve and investigate the nonlinear system of differential equations. Exact solutions are obtained for the electrostatic potential and velocity. Different from the work of Niazi and Xu [17], we have considered a partially filled microchannel with a porous medium and used the stress jump condition at the interface. Two momentum boundary layers are formed in the porous region, to ensure uniform permeability of the porous medium, as illustrated graphically. One of these boundary layers forms immediately adjacent to the impermeable boundary, while the other forms immediately adjacent to the interface. The fluid velocity is constant between these momentum boundary layers. For this reason, the Darcy number and stress jump coefficient show significant variations with velocity, temperature, and nanoparticle concentration. In addition, increasing the physical ratio, i.e., viscosity, decreases the velocity and temperature profiles. Simultaneously, an adverse trend is observed in the volume fraction profile of the nanoparticles. Additionally, the effect of the Darcy number and stress jump coefficient on the skin friction, and the Nusselt number on the upper and lower microchannel walls, are visible. As a result, it is concluded that the stress jump boundary condition is critical for solving fluid flow problems in a wide variety of practical applications.

## Figures and Tables

**Table 1:** Numeric values for the physical attributes of the fluid and nanoparticles.

Physical Characteristic	$H_{2}O$	${\mathrm{Al}}_{2}O_{3}$	${\mathrm{TiO}}_{2}$
$c_{p}\;\left(J\cdot {\mathrm{kg}}^{-1}\cdot K^{-1}\right)$	4179.0	765.0	686.2
$\rho \;(\mathrm{kg}\cdot m^{-3})$	997.1	3970.0	4250.0
$k\;(W\cdot m^{-1}\cdot K^{-1})$	0.6130	40.0	8.9538
$\alpha \times 10^{-7}\;\left(m^{2}\cdot s^{-1}\right)$	1.47	131.70	30.70
$\beta \times 10^{-5}\;\left(K^{-1}\right)$	21.00	0.85	0.90

## Data Availability

The data presented in this study are available on request. The data include the MATLAB code and the figures.

## Nomenclature

The following abbreviations are used in this manuscript:

${\bar{C}}_{1},{\bar{C}}_{2}$	volumetric fractions of nanoparticles;
$C_{f1},C_{f2}$	local skin friction coefficients;
$D_{B_{1}},D_{B_{2}}$	Brownian diffusion coefficients;
$D_{T_{1}},D_{T_{2}}$	thermophoretic diffusion coefficients;
$\bar{P}$	pressure, Pa;
${\bar{T}}_{1},{\bar{T}}_{2}$	non-dimensional nanofluid temperatures in two regions, *K*;
$V_{1},V_{2}$	non-dimensional velocities of the fluid, m/s;
$Br_{1},Br_{2}$	Brinkman numbers;
$B_{0}$	magnetic field in z-direction;
Da	Darcy number;
${\left(c_{p}\right)}_{f},{\left(c_{p}\right)}_{s}$	fluid and nanoparticle specific heats;
*e*	charge of a proton;
$C_{0}$	reference volume fraction for nanoparticles;
$C_{w}$	volume fraction for nanoparticles on the microchannel walls;
$E_{s}$	non-dimensional external electric field parameter;
$E_{x},E_{y}$	electric field in x− and y−directions, respectively;
$F_{1},F_{2}$	body forces caused by uniform electromagnetic field;
*H*	channel height;
$H_{1},H_{2}$	channel height of two regions;
$h_{1},h_{2}$	non-dimensional heights of two regions;
$Ha_{1},Ha_{2}$	Hartman numbers;
$k_{B}$	Boltzmann constant;
$k_{f1},k_{f2}$	fluid’s thermal conductivity in two regions;
$k_{nf}$	the ratio of the fluid’s thermal conductivities;
*L*	microchannel length;
$n_{0}$	bulk ionic concentration;
$N_{B1},N_{B2}$	Brownian motion parameters;
$N_{T1},N_{T2}$	thermophoresis parameters;
$Nu_{1},Nu_{2}$	local Nusselt numbers;
*P*	non-dimensional pressure gradient;
$q_{w1},q_{w2}$	heat flux on the channel walls;
$Re_{1},Re_{2}$	Reynolds numbers;
$S_{e1},S_{e2}$	lateral direction electric field strengths;
$U_{a1},U_{a2}$	average velocities of the fluid;
$u_{1},u_{2}$	velocity of fluid in two regions;
*W*	microchannel’s width;
x,y,z	Cartesian coordinates;
$\hat{z}$	ion valency.
GreekLetters	
$\alpha_{1},\alpha_{2}$	thermal diffusivity of the nanofluid, $m^{2}s^{-1}$;
ϵ	porosity of the porous region;
$\varepsilon_{0}$	permittivity of vacuum, mkgs;
$\varepsilon_{R_{1}},\varepsilon_{R_{2}}$	medium’s dielectric constants;
ε,	medium’s dielectric constant ratio;
κ	permeability of the porous region;
γ	constant coefficient;
β	the adjustable stress jump coefficient;
${\bar{\psi }}_{1},{\bar{\psi }}_{2}$	dimensional electrostatic potential, *V*;
${\left(\rho_{n}\right)}_{s}$	nanoparticles density, $kgm^{-3}$;
${\left(\rho_{n}\right)}_{f}$	nanofluid’s density, $kgm^{-3}$;
${\bar{\rho }}_{e_{1}},{\bar{\rho }}_{e_{2}}$	densities of charges, $Cm^{-3}$;
η	non-dimensional spatial variable;
$\Gamma_{1},\Gamma_{2}$	non-dimensional pressure gradient parameters;
$k_{1},k_{2}$	electro-osmotic parameters;
$\lambda_{N}$	the ratio of the any physical quantity *N*, where $N\in \varepsilon ,nf,\mu ,\sigma ,D_{B},D_{T},\alpha ,\tau ,\rho$;
$\mu_{1},\mu_{2}$	fluid’s dynamic viscosity in two regions;
$\mu_{eff}$	the ratio of viscosity to porosity in Region II;
$\theta_{0}$	dimensionless reference temperature;
$\theta_{1},\theta_{2}$	temperature distributions (non-dimensional);
$\Psi_{1},\Psi_{2}$	non-dimensional nano-particle volume fractions;
${\bar{\rho }}_{e1},{\bar{\rho }}_{e2}$	densities of the charges;
$\tau_{w1},\tau_{w2}$	shear stresses on channel’s opposite walls;
$\psi_{1},\psi_{2}$	non-dimensional nano-particle volume fractions;
${\bar{\zeta }}_{1},{\bar{\zeta }}_{2}$	zeta potentials (dimensional).
$\zeta_{1},\zeta_{2}$	zeta potentials (non-dimensional);
$\Phi_{1},\Phi_{2}$	viscous dissipation factors;
SubscriptsIndices	
1,2	indices for regions I and II;
s,f	subscript notations for solids and fluids;
*w*	indicate the quantities on walls of the channel.
Abbreviations	
EDL	electric double layer;
FDM	finite difference method.
